# HCV-Associated Nephropathies in the Era of Direct Acting Antiviral Agents

**DOI:** 10.3389/fmed.2019.00020

**Published:** 2019-02-08

**Authors:** Andrea Angeletti, Chiara Cantarelli, Paolo Cravedi

**Affiliations:** ^1^Nephrology Dialysis and Renal Transplantation Unit, S. Orsola University Hospital, Bologna, Italy; ^2^Division of Nephrology, Department of Medicine, Icahn School of Medicine at Mount Sinai, New York, NY, United States

**Keywords:** direct acting antivirals, HCV, cryoglobulinemia, rituximab, kidney transplant

## Abstract

Hepatitis C virus (HCV) infection is a systemic disorder that frequently associates with extrahepatic manifestations, including nephropathies. Cryoglobulinemia is a typical extrahepatic manifestation of HCV infection that often involves kidneys with a histological pattern of membranoproliferative glomerulonephritis. Other, less common renal diseases related to HCV infection include membranous nephropathy, focal segmental glomerulosclerosis, IgA nephropathy, fibrillary and immunotactoid glomerulopathy. Over the last decades, the advent of direct-acting antiviral therapies has revolutionized treatment of HCV infection, dramatically increasing the rates of viral clearance. In patients where antiviral therapy alone fails to induce renal disease remission add-on B-cell depleting agents represent an alternative to counteract the synthesis of pathogenic antibodies. Immunosuppressive therapies, such as steroids, alkylating agents, and plasma exchanges, may still represent an effective option to inhibit immune-complex driven inflammatory response, but the potentially associated increase of HCV replication and worsening of liver disease represent a serious limitation to their use.

## Introduction

Hepatitis C virus (HCV), first identified in 1989, is an enveloped positive-stranded RNA virus belonging to the Flaviviridae family (1). HCV is a globally prevalent pathogen and a major health concern. According to the World Health Organization (WHO), in 2015, 71 million people had chronic HCV infection (estimated global prevalence of 1%), putting these individuals at high risk for progressive liver disease including cirrhosis and hepatocellular cancer. HCV infection is generally asymptomatic and only 20–40% of individuals clear the virus spontaneously, therefore most of the subjects that encounter the virus become chronically infected (2). HCV-associated all-cause mortality is double compared to HCV-negative individuals and extrahepatic manifestations represent a major risk factor (3). Lymphoproliferative and autoimmune disorders, ranging from cryoglobulinemia vasculitis to malignant B-cell lymphoma, are the most common extrahepatic conditions associated with HCV infection (4). Large cohort studies have revealed additional extrahepatic manifestations, including cardiovascular, neurological, metabolic, and renal conditions (5) and multiple manifestations often coexist in the same patient. Cacoub et al. (6) have reported that up to 74% of chronically HCV infected patients suffer from at least one extrahepatic manifestation.

Several multi-center survey studies have reported the epidemiology of HCV infection in individuals with end stage renal disease (ESRD): according to the Dialysis Outcomes and Practice Patterns Study (DOPPS) (7), a large observational study including 49,762 ESRD subjects in 12 developed countries, the prevalence of anti-HCV antibody positivity is 9.5%. In developing countries, the prevalence of HCV infections among ESRD patients is less clear and ranges across different reports between 6.1 and 49.6% (8).

In renal transplant recipients, the prevalence of HCV infection varies from 6 to 46% (9) and in most cases the infection occurs before transplant rather than through an infected donor (10). Several studies reported that 74 to 92% of HCV positive renal transplant recipients have detectable HCV RNA levels at the time of transplantation, which persist (11) and rise after antirejection therapy is initiated (12). HCV infection associates with shorter graft and patient survival (13) and renal transplantation increases the risk to develop hepatocellular carcinoma in HCV infected patients (14).

The treatment options for HCV infection have markedly expanded, with a dramatic acceleration since 2001, when the interferon-based regimens were first integrated with and then replaced by direct-acting antiviral drugs (DAAs) (15). Based on the excellent results obtained with the new anti-HCV therapies, one of the goals of the United Nations 2030 Agenda for Sustainable Development is the removal of viral hepatitis as a threat for the public health, with targets including an 80% reduction in the incidence of HCV infections and a 65% decrease in HCV-related mortality (16).

## HCV-Associated Nephropathies

Renal impairment in chronic HCV infection is mostly related to mixed cryoglobulinemia, a systemic vasculitis that mainly affects small-sized vessels and that in the kidney generally leads to membranoproliferative glomerulonephritis (MPGN).

Other glomerular diseases that have been associated with HCV infection include membranous nephropathy, focal segmental glomerulosclerosis (FSGS), fibrillary, or immunotactoid glomerulopathy, and IgA nephropathy (17). Typical renal manifestations in HCV-infected patients include proteinuria, microscopic hematuria, hypertension, and nephrotic syndrome and the triad of purpura, asthenia, and arthralgia is evident in nearly 30% of the cases (18).

HCV evolved in 7 different genotypes and more than 67 subtypes. A community-based prospective study (19) involving 13,805 participants showed an association between HCV genotype 2 and chronic kidney disease. Differently, the REVEAL-HCV study involving 19,984 participants (20), showed that HCV genotype 1 and high serum HCV RNA levels (>167,000 IU/mL) are strong predictors of ESRD. While the impact of the different HCV genotypes on renal outcomes still needs to be completely elucidated, careful renal function evaluation should be part of regular follow-up of individuals with HCV infection, especially if serum levels of HCV RNA are elevated and in case of infection with HCV genotypes 1 or 2. Moreover, in last decade several genome-wide association studies reported many host genetic factors that influence hepatic outcomes and treatment efficacy after HCV infection (21–23). A GWAS among patients with chronic HCV infection found a genome-wide significant association of rs9461776 (*HLA-DRB1/DQA1*) with cryoglobulin-related vasculitis (24) The same study also identified single nucleotide polymorphisms (SNPs) near *NOTCH4* and *MHC* class II that were strongly associated with this syndrome.

### Cryoglobulinemic Glomerulonephritis

Cryoglobulins are defined as polyclonal immunoglobulin G (IgG) bound to another immunoglobulin that acts as anti-IgG rheumatoid factor, that together precipitate in serum cooled to 4°C. According to Brouet et al. (25), the cryoglobulins can be subdivided into three subgroups: type I contains an isolated monoclonal immunoglobulin, type II comprises IgG and an IgM rheumatoid factor (RF) of monoclonal origin (previously called mixed essential cryoglobulinemia), and type III comprises IgG and a polyclonal IgM RF. Cryoglobulins associated with HCV infection are of type II (26), while type I cryoglobulins are associated with lymphoproliferative disorders (27) and type III cryoglobulins are often related with connective tissue diseases, infections, hepatobiliary diseases, and lymphoproliferative disorders (28).

**Figure 1:**
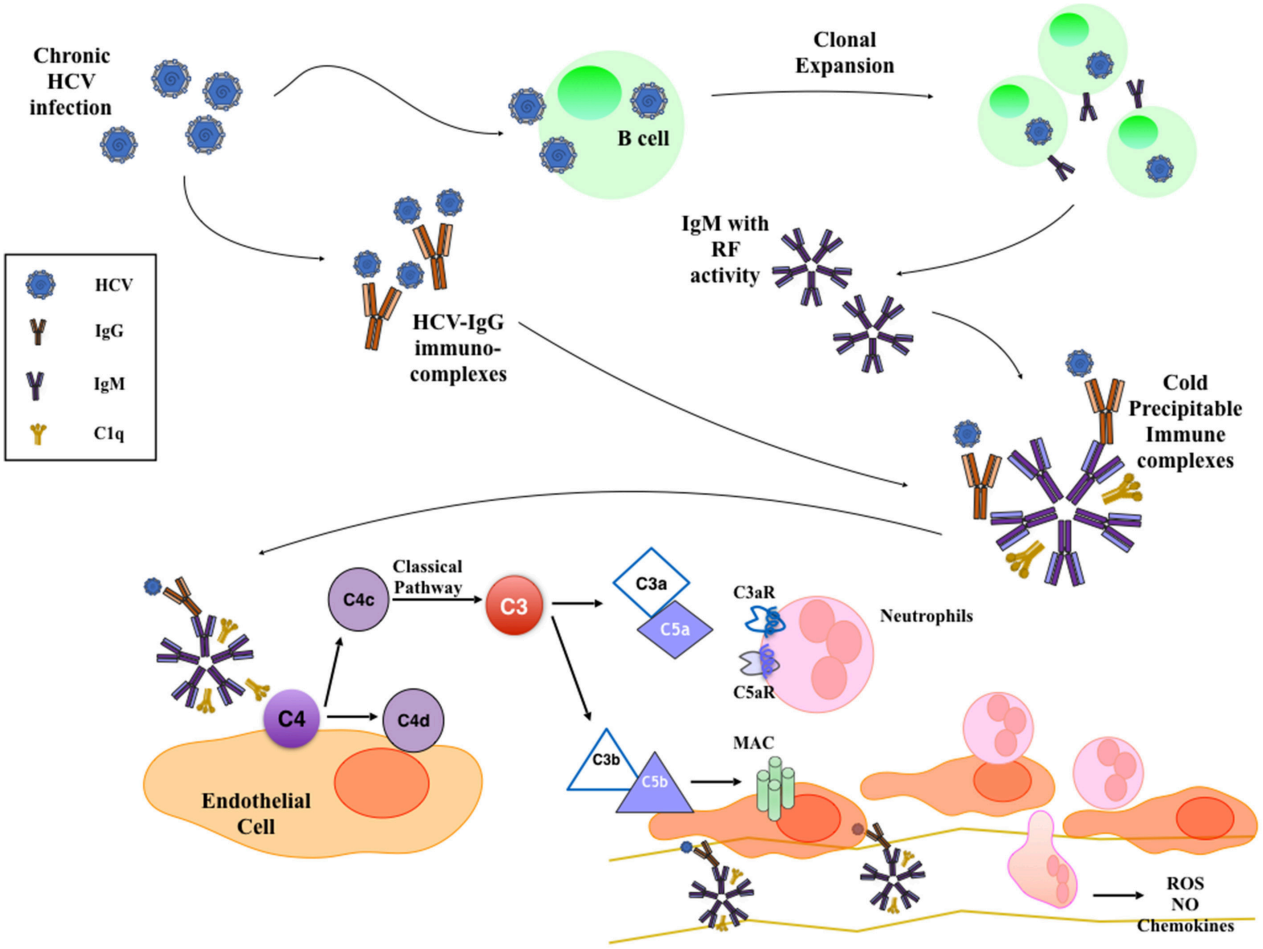
Mechanism of HCV-Induced Cryoglobulinemic Nephropathy. HCV infection of B cells leads to the production of IgM with rheumatoid factor (RF) activity that bind HCV-IgG immune-complexes. These cold-precipitable multimolecular immune-complexes deposit in the subendothelial space and in the mesangium, where they activate classical complement pathway. This leads to the formation of C3a and C5a anaphylatoxins that recruit and activate inflammatory cells and to the deposition of membrane attack complex (MAC) on the endothelium that activates endothelial cell proinflammatory functions.

Cryoglobulinemic glomerulonephritis is caused by cryoglobulin deposits in the glomerular capillary walls (often in the subendothelial space) and in the mesangium, giving an MPGN pattern of injury (29, 30) (Figure 1). The clinical presentation includes hypertension, proteinuria, microscopic hematuria, acute nephritis, or nephrotic syndrome, often associated with C3 and/or C4 complement consumption. All three types of cryoglobulins, including those due to monoclonal or polyclonal immunoglobulins, can cause cryoglobulinemic GN, but it occurs most often with HCV-associated type II cryoglobulinemia (Table 1). Until recent treatment advances, HCV-associated MC was associated with 1-, 3-, 5-, and 10-year survival rates of 96, 86, 75, and 63%, respectively (31).

**Table 1:** Pathogenic mechanisms of kidney injury in HCV infection related nephropathies.

**HCV-related nephropathy**	**Cryoglobulin type**	**Histological phenotype**	**Mechanism of kidney injury**
Cryoglobulinemic GN	Type I: isolated monoclonal IgA, IgM, or IgG Type II: IgG and a monoclonal IgM rheumatoid factor Type III: IgG and a polyclonal IgM rheumatoid factor	Membranoproliferative GN (most frequently associated with type II cryoglobulinemia)	IC deposition in: -the lumen of glomerular capillaries (eosinophilic thrombi) -the subendothelium of capillary walls with endothelitis by complement activation -the mesangium, due to their high affinity for fibronectin in the mesangial matrix Impaired clearance of ICs by monocytes and macrophages.
Non-cryoglobulinemic GN		Membranoproliferative GN	Mesangial deposition of IC with viral-like particles, IgG and complement fractions
		Membranous nephropathy	Subepithelial glomerular deposition of IC containing HCV proteins
		IgA nephropathy	Impaired IgA clearance and IgA-containing IC
		Focal segmental glomerulosclerosis	Possible direct injury of podocytes induced by HCV
		Fibrillary and immunotactoid glomerulopathy	Extracellular deposits of microfibrils within the mesangium and glomerular capillary walls
			IgG4 predominance in the deposits, like in other fibrillar GN

*GN, glomerulonephritis; IC, immunocomplexes; Ig, immunoglobulin; HCV, hepatitis C virus; RF, rheumatoid factor*.

Histological appearance at light microscopy shows mesangial proliferation and often diffuse and global endocapillary hypercellularity. Cryoglobulins can also deposit in glomerular capillaries as eosinophilic thrombi that usually associate with vasculitis and fibrinoid necrosis of glomeruli. The acute phase often shows neutrophils, while monocyte/macrophages infiltrate in both acute and chronic stages (Figure 1). Arterioles and small arteries may show leukocytoclastic vasculitis, sometimes with cryoglobulin deposits (32). With severe glomerular inflammation and damage of the glomerular capillary wall, cryoglobulins can lead to extracapillary proliferation and crescent formation (33).

At immunofluorescence, capillary walls show significant IgM and C3 staining. Intracapillary thrombi are typically positive for IgM and clonal κ or λ chain staining. Electron microscopy shows subendothelial and mesangial dense deposits, usually with interposed cells and double contours due to new GBM formation beneath subendothelial deposits. Moreover, considerable effacement of podocyte foot processes and endocapillary hypercellularity are often reported (Table 1) (32). Several studies investigated the interaction between HCV and the complement system, establishing an active role of complement in intra- and extrahepatic manifestations of HCV infection. Similarly to autoantibody-initiated kidney glomerulopathies, complement activation in type II cryoglobulinemia occurs prevalently through the classical pathway and promotes injury through the recruitment of inflammatory cells and membrane attack complex formation (34–36).

### Other HCV-Associated Nephropathies

Cases of membranous nephropathy have been reported in patients with HCV-infection (37), with clinical presentation and histological findings that are similar to primary forms. Yamabe et al. (38) found that 8.3% of membranous nephropathy patients had anti-HCV-antibodies or detectable HCV RNA. The pathogenesis of membranous nephropathy in HCV infected patients is thought to be related to the deposition of immune complexes containing HCV proteins in glomeruli, where viral-like particles have been identified by electron microscopy (39). Glomerular deposition of IgM, IgG, IgA, and complement with the same distribution of HCV (40) strongly support this hypothesis (33, 41).

Fibrillary-immunotactoid glomerulopathy was described in few HCV-infected individuals. It is characterized by extracellular mesangial deposits of microfibrils, positive staining of glomerular capillary walls for IgG4 and C3 (17, 42, 43) and negative for Congo red staining (37). Fibrillary-immunotactoid glomerulopathy typically manifests with clinical and laboratory signs of nephritic syndrome (hematuria, hypertension, and renal failure), but with proteinuria in nephrotic range (44–46).

HCV infection has also been associated with IgA nephropathy (47, 48). Pathogenic link between the two conditions is supported by the evidence that antiviral therapy with IFN-α leads to renal disease remission (49–51). However, due to the reduced hepatic clearance of IgA and IgA-containing immune complexes, IgA deposition in the glomeruli are common in all forms of cirrhosis. Therefore, HCV may not be directly implicated in pathogenesis of the disease (Table 1) (52, 53).

### HCV-Associated FSGS

Glomerular lesions associated with HCV infection are mostly sustained by cryoglobulins and immune complex deposits. However, antibody-independent glomerulonephritides have also been described in HCV positive patients that may display features of FSGS. While the pathogenic mechanisms are unclear, it is hypothesized that, similar to human immunodeficiency virus (HIV), HCV directly injures podocytes, leading to glomerulosclerosis (54).

## HCV Infection in Kidney Transplant Recipients

In kidney transplant recipients, HCV infection is associated with increased morbidity and mortality rates, due to hepatic and extra-hepatic complications (55–57). A retrospective study of 706 HCV positive renal transplant recipients found that the presence of HCV antibodies independently predicted reduced patient and graft survival at 10 years. There is no clear evidence of an association between the kind of antirejection therapy and HCV activity in kidney transplant recipients. Some reports suggest a potential benefit of mTOR inhibitors in controlling viral replication (58–60), but this therapeutic approach should be weighed against the increased risk of acute rejections and the poor tolerability of these agents (61).

Extra-hepatic disease associated with HCV infection in transplant recipients includes *de novo* or recurrence of glomerular diseases, acute rejection, transplant glomerulopathy, and accelerated kidney graft fibrosis (62). MPGN is the most common glomerulopathy in HCV-infected kidney transplant recipients that occurs in 5–54% of patients (63). The presence of anti-HCV antibodies before kidney transplantation is a risk factor for the occurrence of proteinuria and reduced graft survival (64).

Co-infection with HIV seems to be an independent risk factor for graft failure and patient survival compared to HCV infection alone (65). As recently showed by Rallòn et al. (66), HCV related immune defects accelarate HIV disease progression, supporting early anti-HCV treatment in case of combined HIV/HCV infection.

## Therapies for HCV-Associated Nephropathies

A better understanding of the pathophysiology of HCV-associated nephropathies has progressively opened the door to more targeted, hypothesis-driven approaches: (a) antiviral treatment to avoid the formation of cryoglobulins, immune complexes and direct viral injury to the kidney; (b) B-cell depletion, aimed at reducing cryoglobulin production, and (c) immunosuppressive treatments targeting glomerular inflammation.

### Antiviral Agents

Differently from HBV and HIV, HCV infection can be completely and permanently cured by antiviral treatment as HCV has no long-term reservoir in the body. The definitive cure of HCV infection is commonly reflected by the sustained virologic response (SVR), defined as no-viremia for 24 weeks after ending antiviral therapy. Attaining an SVR has been associated with decreased all-cause mortality and need for liver transplantation, even among patients with advanced liver fibrosis (67, 68).

Interferon and ribavirin still represent the standard of care for recent HCV infection, but the management of subjects with chronic infection has been revolutionized by the development of HCV-specific antiviral drugs (direct acting antivirals–DAAs). HCV-encoded proteins (NS3/4A protease, NS5A protein, and NS5B polymerase) are fundamental for virus replication and represent the main target of the DAAs [for more details see (69)]. Combining two or more DAAs from different classes has increased SVR from ~50% (70) to over 90% (71) and shortened treatment duration to only 8–12 weeks in most populations with chronic HCV infection (71–75).

According to the 2015 guidelines by the American Association for the Study of Liver Diseases (AASLD) and the Infectious Diseases Society of America (IDSA) (76), all of the approved DAA regimens can be used in patients with estimated glomerular filtration rate (eGFR) >30 ml/min/1.73 m^2^ (77). In subjects with eGFR < 30 ml/min/1.73 m^2^ or in individuals on dialysis, the three approved regimens are: (1) ritonavir-boosted paritaprevir, ombitasvir, and dasabuvir (2) ribavirin, elbasvir and grazoprevir, and (3) glecaprevir + pibrentasvir (78–81).

### DAAs in the Treatment of MC-GN

Reports on DAAs treatments in subjects with HCV-GN are limited. Saadoun and colleagues. (82) treated five HCV-MC subjects with sofosbuvir and RBV for 24 weeks and showed that eGFR increased, while proteinuria declined in four cases. Similarly, Sise et al. (83) reported that four out of seven HCV-positive patients with renal impairment had a reduction of proteinuria and/or eGFR increase after sofosbuvir-based treatment. More recently, Saadoun et al. (84) showed that sofosbuvir and daclatasvir induced complete remission in five patients with renal involvement. Collectively, over 60% of the studies that evaluated response of MC-GN patients to DAAs treatment, showed complete resolution of GN or improvement in renal parameters (Table 2) (82–93). Despite DAAs treatment in MC-GN implies higher medication costs compared to classical treatments, Cacoub et al. (94) have elegantly demonstrated how costs related to hospitalizations and supportive therapies decreased. The cost/effectiveness of DAAs therapies may become more advantageous in the future, in light of recent indications for reducing DAAs treatment durations and lowering price (95).

**Table 2:** Major published clinical studies of antiviral and immunosuppressive treatments for HCV-related glomerulonephritis.

**Reference**	***N***	**Study** **design**	**With Kidney disease *(N)***	**HCV** **drugs**	**On Immuno- suppressants *(N)***	Complete renal response* (%)	Partial Renal response^*^ (%)	**SVR (%)**	**Comments**
Saadoun et al. (93)	41	Prospective cohort	5 (MPGN: 4)	SOF + DAC	0	80	20	100	Proteinuria decreased from 0.9 ± 0.4 to 0.2 ± 0.1 g/24 h.
Emeryet al. (91)	83	Prospective cohort	10	SOF + RBV/SIM, RBV +3 D, SOF + LDV ± RBV	RTX (3) PE (4)	na	na	na	Four patients presented with life-threatening vasculitis and two of them required dialysis.
Bonacci et al. (89)	64	Prospective cohort	10	SOF + LDV/SIM/DAC, SIM + DAC	RTX (3) PE (1)	70	29	100	All patients with renal involvement achieved SVR12, and seven of them also had a complete renal response.
Gragnani et al. (88)	44	Prospective cohort	4 (MPGN: 1)	SOF + DAA	RTX (1)	75	25	100	One patient had nephrotic syndrome and received albumin and diuretics; 3 patients had reduced GFR and proteinuria.
Sise et al. (83)	12	Case series	7 (MPGN: 5)	SOF + SIM (6) /RBV(1)	RTX (1)	43	57	85.7	Combined RTX, steroids, and plasmapheresis were used in six patients before initiation of DAAs.
Saadoun et al. (84)	41	Prospective cohort	5 (MPGN: 4)	SOF + RBV	RTX (2)	0	80	80	Proteinuria decreased from 1.09 (0.6–2.4) to 0.17 (0.07–0.25) g/24h.
Saadoun et al. (82)	23	Prospective cohort	7	IFN + RBV + BOC /TVR	RTX (4)	71	29	na	RTX did not affect the rate of virological response.
Cornella et al. (87)	5	Case series	5 (MPGN: 2)	IFN + RBV + BOC/TVR (2), IFN+SOF+RBV (1)	0	66	0	100	Two patients were treated with RTX before starting therapy for HCV.
De Vita et al. (85)	57	RCT	17	0	RTX (7) vs. non RTX (10)° steroids AZA or CYC PE	29	29	na	RTX therapy associated with 3.5 higher risk of proteinuria remission.
Sneller et al. (86)	24	RCT	8	0	RTX (4) *vs* non RTX (4)° steroids CYC	0	100	100	RTX treatment was associated with stable renal function, while the control group experienced GFR decline in the over the 6-month follow-up period.

* *Defined as proteinuria and/or haematuria absent and/or growth of glomerular filtration rate (GFR)*.

° *One of the listed treatments*.

*3D, ombitasvir-paritaprevir-ritonavir and dasabuvir; BOC, boceprevir; CYC, cyclophosphamide; DAA, direct acting antivirals; DAC, daclatasvir; GFR, glomerular filtration rate; IFN, interferon; LDV, ledipasvir; MPGN, membrano-proliferative glomerulonephritis; na, not available; RBV, ribavirin; RCT, randomized clinical trial; RTX, rituximab; SIM , simeprevir; SOF, sofosbuvir; SVR, sustained virologic response; TPE, therapeutic plasma exchange; TVR, telaprevir*.

### B-Cell Depletion

Clearing the viral trigger of HCV-associated glomerular diseases is the ideal treatment for MC-GN. However, B-cell depleting therapies are also widely used, either alone or in combination with antiviral therapies, to prevent immune complex formation and control disease progression. Rituximab is a chimeric monoclonal depleting antibody targeting the CD20 antigen expressed on B cells (96). Rituximab depletes naïve and memory B cells through the induction of apoptosis, antibody-dependent cell-mediated cytotoxicity, phagocytosis and complement-mediated cytotoxicity (97).

In a single-center, open-label trial, 24 patients with HCV-associated cryoglobulinemic vasculitis in whom antiviral therapy had failed to induce remission, were randomized to immunosuppressive therapy + rituximab or immunosuppressive therapy alone (Table 2). After 6 months, ten patients in the rituximab group and only one in the non-rituximab group were in remission (86). In a multicenter, phase III, randomized controlled trial (85), 59 patients with cryoglobulinemic vasculitis (associated to HCV infection in 53 cases) were allocated to rituximab vs. immunosuppressive treatment (glucocorticoids with azathioprine, cyclophosphamide, or plasmapheresis). Among the 16 patients with glomerulonephritis, four out of seven of those in the rituximab group had complete or partial response, while none of those in the non-rituximab group reported a significative reduction in proteinuria.

Rituximab has also been tested as add-on to anti-viral therapy (Table 2). A prospective controlled trial showed a higher rate of complete kidney response (81 vs. 40%) and a good safety profile in 31 patients with severe HCV-associated cryoglobulinemic vasculitis randomized to rituximab plus PEG-IFN/RBV combined therapy vs. PEG-IFN/RBV alone therapy (98). Similar data were obtained in the subset of 9 patients with kidney involvement (99). In line with the above studies, other reports showed the safety of rituximab in HCV-infected individuals, which have shown no increase in HCV viremia and stable liver function tests after rituximab therapy (86, 98) (Table 2).

### Non-specific Immunosuppressive Therapy

Plasma exchange has been considered for years the treatment of choice for subjects with cryoglobulinemic vasculitis, with or without renal involvement, with the aim of removing circulating cryoglobulins and preventing the deposition of immune complexes (100). Cyclophosphamide has also been used to suppress B cell function and cryoglobulins production, but this treatment should be used with caution because it can induce flare-ups of HCV infection (101). Compared to cyclophosphamide, mycophenolate mofetil is a more selective treatment to inhibit lymphocyte proliferation and function and represents a safer alternative to induce remission in cryoglobulinemic vasculitis (102). Steroid pulses or low doses of steroids have been used to treat glomerular infiltration (100), but steroids may favor HCV replication and worsen liver disease (37). Due to the lack of strong evidence-based recommendations for the treatment of HCV-related glomerular disease, plasma exchange and conventional immunosuppression still represent an option before starting specific HCV antiviral treatment.

## Treating HCV in Patients on Renal Replacement Therapies

According to the DOPPS study (103), <2% of HCV-positive ESRD patients and < 5% of those wait-listed for renal transplant receive treatments for HCV eradication. The main reason for not treating these patients before transplant is to provide them with the opportunity to obtain kidneys from HCV-positive donors, which may reduce the time on the waiting-list. This could be a reasonable approach in countries such as the United States, where HCV-positive donors represent more than 20% of the donor pool (104) and are often young and with limited comorbidities (105). On the contrary, in European countries, where percentage of HCV positive donors is more limited, this theoretical advantage would be lost (57) and treatment of HCV patients in dialysis should not be further delayed.

Importantly, preliminary data indicate that DAAs therapy may provide the opportunity to allocate renal transplant from HCV-positive to HCV-negative subjects. In the THINKER (Transplanting Hepatitis C Kidneys into Negative Kidney Recipients) trial (106), a 12-week regimen of elbasvir/grazoprevir achieved SVR and renal function improvement in 10 HCV-negative subjects receiving kidneys from HCV-positive donors. Despite these and other encouraging results (107), the evidence on the safety/efficacy profile of DAAs treatment in patients on renal replacement therapies is limited and does not support its widespread use.

Available data regarding the treatment of HCV positive kidney transplant recipients with DAAs are still few, but several reports support a favorable safety/efficacy profile and available evidence indicates that such therapies do not affect the risk of acute rejection (108). Recent studies demonstrate that DAAs therapies effectively cured HCV in 406 of 418 kidney transplant recipients (97%) at 12 months, without affecting the risk of acute rejection (108). However, in a multicenter trial, Colombo et al. (109) randomized 114 kidney transplant recipients with HCV infection to a 12- or 24-week course of ledipasvir/sofosbuvir. All patients achieved SVR (primary endpoint) at 12 and 24 weeks, respectively, and treatment-related reduction of eGFR was reported in three patients, but with no evidence of acute rejection.

## Conclusions

Chronic HCV infection affects more than 170 million people worldwide and is responsible for over 350,000 deaths every year. Besides chronic liver disease, HCV infection is associated with extrahepatic manifestations including cryoglobulinemia, lymphoproliferative disorders, and renal diseases.

HCV is associated with a large spectrum of renal lesions and clinical sequelae, in both native and transplanted kidneys. The most common HCV-associated renal disease is cryoglobulinemic glomerulopathy, but HCV may associate also with membranous, IgA, and fibrillary nephropathy, amongst others.

Recent data from relatively small studies show promise of the novel antiviral therapies in HCV-associated glomerulopathies. These treatments, together with B-cell depleting agents, may improve outcomes of affected patients. However, the still limited number of studies in this area prevents a clear assessment of the safety/efficacy profile of these treatments.

## Author Contributions

AA, CC, and PC conceived the article contents, prepared the manuscript, and endorsed the final draft submitted.

### Conflict of Interest Statement

The authors declare that the research was conducted in the absence of any commercial or financial relationships that could be construed as a potential conflict of interest.
